# Surface mediated cooperative interactions of drugs enhance mechanical forces for antibiotic action

**DOI:** 10.1038/srep41206

**Published:** 2017-02-03

**Authors:** Joseph W. Ndieyira, Joe Bailey, Samadhan B. Patil, Manuel Vögtli, Matthew A. Cooper, Chris Abell, Rachel A. McKendry, Gabriel Aeppli

**Affiliations:** 1 Departments of Medicine, UCL Institute for Liver and Digestive Health, Royal Free Hospital, London NW3 2QG, UK; 2 London Centre for Nanotechnology and Departments of Medicine and Physics, University College London, 17-19 Gordon Street, London, WC1H 0AH, United Kingdom; 3 Department of Chemistry, Jomo Kenyatta University of Agriculture and Technology, Po Box 62000, Nairobi, Kenya; 4 Centre for Mathematics and Physics in the Life Sciences and Experimental Biology, University College London, 17-19 Gordon Street, London, WC1H 0AH, United Kingdom; 5 Institute for Molecular Bioscience, University of Queensland, Brisbane, 4072, Australia; 6 Department of Chemistry, Lensfield Road, University of Cambridge, Cambridge CB2 1EW, United Kingdom; 7 Laboratory for Solid State Physics, ETH Zurich, Zurich, CH-8093, Switzerland; 8 Institut de Physique, EPF Lausanne, Lausanne, CH-1015, Switzerland; 9 Photon Science Division, Paul Scherrer Institut, Villigen PSI, CH-5232, Switzerland

## Abstract

The alarming increase of pathogenic bacteria that are resistant to multiple antibiotics is now recognized as a major health issue fuelling demand for new drugs. Bacterial resistance is often caused by molecular changes at the bacterial surface, which alter the nature of specific drug-target interactions. Here, we identify a novel mechanism by which drug-target interactions in resistant bacteria can be enhanced. We examined the surface forces generated by four antibiotics; vancomycin, ristomycin, chloroeremomycin and oritavancin against drug-susceptible and drug-resistant targets on a cantilever and demonstrated significant differences in mechanical response when drug-resistant targets are challenged with different antibiotics although no significant differences were observed when using susceptible targets. Remarkably, the binding affinity for oritavancin against drug-resistant targets (70 nM) was found to be 11,000 times stronger than for vancomycin (800 μM), a powerful antibiotic used as the last resort treatment for streptococcal and staphylococcal bacteria including methicillin-resistant *Staphylococcus aureus* (MRSA). Using an exactly solvable model, which takes into account the solvent and membrane effects, we demonstrate that drug-target interactions are strengthened by pronounced polyvalent interactions catalyzed by the surface itself. These findings further enhance our understanding of antibiotic mode of action and will enable development of more effective therapies.

While molecular recognition exhibits complementarities between a host and guest, cross-reactive binding at a single docking site is possible^1^,^2^. For a binding site to interact with different ligands, binding must be treated as a dynamic process with the population of the ensemble being in equilibrium, and shape of binding sites strongly influenced by the incoming partner^3^. However, in cell-mediated immune response^4^ and antimicrobial activity^5^, the doctrine of molecular selectivity is a prerequisite for ligand-receptor binding interactions. Vancomycin (Van) exemplifies this principle by specifically targeting amino acid residues of peptide domains which are only found in bacteria. Specific drug-target interactions not only inhibit cell wall biosynthesis^6^,^7^ but can also impose mechanical force on the overall cell via cell wall stress changes^8^. Modifications of receptors at the surface of a bacterium cell, however, can alter the selectivity of drug-target interactions in bacteria, thus inactivating the recognition mechanisms and associated mechanical stress. For example, *Enterococcus faecalis, Enterococcus faecium* and *Staphylococcus aureus* are well-known aetiological agents of a wide variety of infections caused by structural changes at a cellular target. Antimicrobial resistance (AMR) in vancomycin-resistant *Staphylococcus aureus* (or VRSA)^9^ is caused by cell wall thickening while for vancomycin-resistant enterococci (or VRE) is conferred by the reprogramming of terminal alanine amino acid residues of bacterium cell^10^.

The alarming increase of pathogenic bacteria that are resistant to multiple antibiotics is now recognized as a major health issue^11^ putting at risk society’s ability to treat common infections. To prevent and control the spread of AMR requires development of new drugs and novel interventions to infections. Since the discovery of penicillin and other antibacterial agents, a large number of studies have greatly enhanced our understanding of how antibiotics induce cell death. Interestingly, in nearly all work on antimicrobial activity^12^ cell death is presumed to be primarily caused by the inhibition of one of a few essential cellular functions such as cell wall biosynthesis, protein synthesis and DNA or RNA signaling. The exploration of bacterial mechanobiology^13^ with the view to developing novel antibacterial therapies has, however, been largely overlooked.

Here, we show that the mechanical forces induced by drug-target interactions, regulated by solvent interactions and membrane effects, are critical to our understanding of bactericidal activity against drug-resistant bacteria. In order to demonstrate that molecular changes within a membrane receptor can incapacitate recognition and efficacy of drugs (Fig. 1a–d), we used two extracellular model targets found in bacterial cell envelopes, herein termed vancomycin-susceptible receptor (or VSR)^14^ and a reprogrammed version of VSR termed vancomycin-resistant receptor (or VRR). While VSR functions as an attractive surface “lock” to sense an antibiotic’s “key”, the VRR motif is less attractive as a “lock” because of the changes in an amide NH group to an ester^15^ which increases the repulsive effects in an oxygen lone pair (Fig. 2). VSR and VRR were therefore used as targets to investigate the impact of a mismatch on the molecular recognition process. To dissect the mechanisms involved in controlling molecular recognition processes and provide solutions to the mechanoselectivity in drug-resistant targets, we formulated for the first time an analytical theory, explicitly including solvent interactions and membrane effects. We propose that confinement of drug molecules to a near-membrane layer can trigger their self-assembly through a process catalysed by the bacterial surface to enable formation of multimeric complexes which is fundamental to the creation of tight drug-target interactions^16^. Specifically, a near-membrane layer formed at the interface between bound receptors and solution, defined at a length of ~1 nm as determined from ionic strength of 0.1 M salt concentration under physiologically relevant conditions, shows that enhanced association kinetics of drugs at the cognate extracellular target is responsible for the surprising efficacy of antibiotics against drug-resistant strains (see definition of near membrane layer, Supplementary Information). We tested this concept on a panel of biologically relevant systems including; Van, the Food and Drug Administration (FDA)-approved drug used as the last resort treatment of MRSA and clostridium difficile infections (CDIs)^17^, oritavancin (Ori or Orbactiv), a recently FDA-approved drug for treatment of complicated skin and soft tissue infections (cSSTIs) as well as ristomycin (Rist) and chloroeremomycin (CE). The latter two drug molecules are not in clinical use for treatment of bacterial infections. These drugs were also tested on the methicillin-susceptible *Staphylococcus aureus* (MSSA) cell line (ATCC 29213).

## Results

### Effect of near-membrane layer on ligand binding efficiency

**Figure 1:**
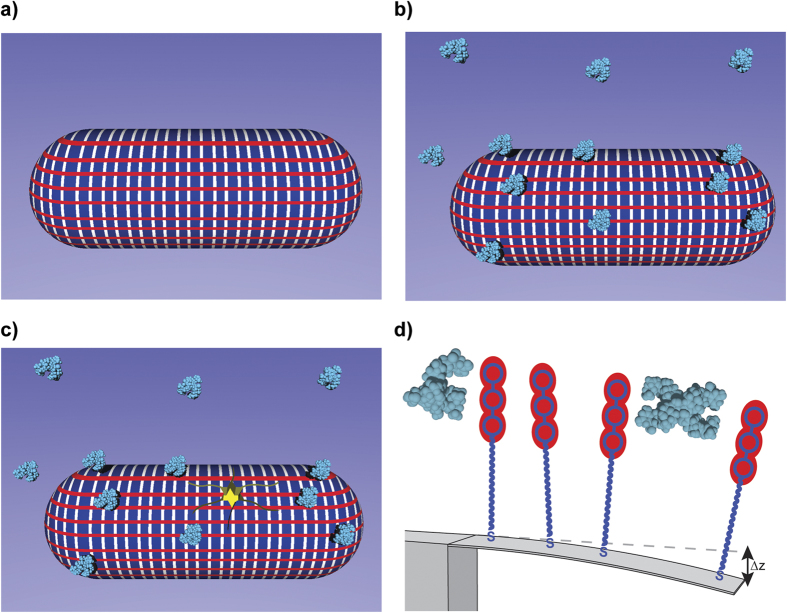
Complex interplay between unrestricted solution phase and membrane targets. (**a**) Schematic representation of Gram-positive bacteria with lipid membrane (blue) surrounded by cell wall composed of peptidoglycan layers anchored to the cell membrane. The peptidoglycan backbone consists of repeat polymers of two amino sugars of N-acetylglucosamine and N-acetylmuramic acid (white solid lines) which are linked to short peptide precursors or bridges (red solid lines). In the absence of antibiotics, the peptide precursors are cross-linked via a peptide bridge with other peptides attached to neighboring glycan chains, resulting into three-dimensional rigid network that is vital for mechanical strength, desired for exoskeletal bacterial function; for clarity, only a two-dimensional network is shown. (**b**) Schematic representation of the corresponding bacterial cell treated with antibiotics (blue chemical structural cartoons) which bind to the peptide precursors (red solid lines) from the glycan backbone to form drug-target complexes that may block rigid network formation and essential cellular activities. (**c**) Schematic representation of possible binding events occurring between antibiotics in solution and membrane bound targets. This is consistent with the picture that following the application of a lethal antibiotic dose, the insertion of drug molecules into bacterium cell wall can induce a local strain which grows as the number of reacted regions grow until a deleterious strain is generated, weakening overall mechanical strength as well as the ability for the cell to counteract high internal osmotic pressure (yielding cracks on the cell wall – yellow depression) which bacteria cannot ultimately withstand, leaving bacterium susceptible to lysis and death. (**d**) Schematic representation of drug molecules binding to a model to bacterial cell wall (red and blue circles), generating stress due to the electrostatic repulsive and steric interactions between bound drug-target complexes (blue chemical structural cartoons between the anchored membrane molecules) causing a cantilever bending deflections, ∆z. This shows the impact of molecular recognition on mechanical signaling.

To probe different pathways of signal-transduction central to the efficacy of recognition mechanisms and therapeutic agents, we used cantilever force-based technology. Because of its nanometre strain sensitivity, this technology can directly measure the smallest changes in mechanical strain caused by specific antibiotic binding. The principles of force transduction and measurements are well established^18^,^19^,^20^ and many studies focused on cantilever sensors have been published^21^,^22^,^23^,^24^,^25^,^26^,^27^. Unlike conventional methods such as 1H–NMR spectroscopy^28^, enzyme-linked immunosorbent assays (ELISA)^29^ or surface plasmon resonance (SPR)^30^ which measure optical signatures of chemical binding, cantilever technology has the capacity to resolve forces at the level of individual hydrogen bonds^31^, making it uniquely suited to provide important insight into force transduction mechanisms, such as cell wall strain in response to antibiotic binding. Moreover, in contrast to direct current or low-frequency signals, which cannot probe beyond electrical double layers formed by screening salts^32^,^33^, mechanical signaling is sensitive to ligands even when they are located at distance of ~1 nm or more from the sensor surface (Fig. 1d). Measurements were performed using cantilevers functionalized with self-assembled monolayers (SAM) of VSR, VRR or reference polyethylene glycol (PEG) (see methods & materials, Supplementary Information). The specificity of mechanical signaling was achieved by performing measurements in differential mode, where the bending response of the reference PEG-coated cantilevers, (known to reduce nonspecific drug adsorption^8^,^14^,^34^) was subtracted from VSR- and VRR-coated cantilever signals (see cantilever measurement procedure, method section).

As proof of concept and to understand how membrane effects regulate molecular recognition to stimulate force transduction and transmission, we investigated the impact of near-membrane rather than bulk solvent effects on the antibiotic binding efficiency. This was achieved by limiting the distance at which the membrane targets are felt by molecules in solution via changing the ionic strength of the medium whilst keeping the solution pH at 7.4 to reflect physiological conditions. Assays were performed under constant flow conditions at three different ionic strengths for the same antibiotic concentrations. While constant flow experiments can induce an offset during mechanical deflection measurements depending on the position of the chip within the flow system, such variations are eliminated if a scanning laser is aligned within the middle region of the position sensitive detector (PSD). Figure 3a shows the outcome after injection of Van against VSR, initially kept constant at 1000 μM to ensure full surface coverage^34^. The results reveal a mechanical response, as measured by the free end deflection, of ~150 nm when near-membrane layer thickness is ~50 nm and ~220 nm for a layer thickness of ~1 nm (Fig. 3a,b). The measurements show that the association kinetics of Van accelerate with decreasing near-membrane layer thickness. This is because when near-membrane distance is reduced the effect of ionic shielding and molecular charge is increased thus permitting close contact between the drug molecules and so triggering attractive forces, such as induced dipole interaction, which are important for occasioning complex aggregation of drug molecules (whose strength is determined by the complex aggregation constant *K*_1_, the equilibrium dissociation constant of ligand-ligand interactions). Conversely, when a near-membrane layer thickness is increased, ionic shielding and molecular charge is reduced, thus hindering molecules from close contact and decreasing the association kinetics^35^. To explain the general principle of the observed mechanical response we hypothesize that prior to the introduction of Van molecules (as shown by grey shaded area in Fig. 3a), receptor molecules interact with each other only weakly, if at all, which explains the minimal or zero mechanical response relative to the reference PEG control. However, the exposure of Van to VSR leads to the formation of Van-VSR complexes, which carry an electrostatic charge^36^. The resulting electrostatic repulsive and steric interactions between Van-VSR complexes induce local strain in the silicon, which creates compressive stress on the Au top surface (Fig. 3a). For the cantilever to bend downwards with the inclusion of bound complex, we propose that the monolayer on top must expand, imparting a tensile stress on the membrane receptor itself.

### Chemical functionality of groups remote from the binding sites explain high affinity required for enhanced force transduction

In order to demonstrate the feasibility of membrane effects in regulating molecular recognition to enhance force transduction, we considered the interaction of drugs at the interface between bacterial surface and solution to be defined by three-dimensional (3-D) rates and affinity constants, while the induced mechanical response following drug binding is restricted to a two-dimensional (2-D) environment (Fig. 1a–d). In the first set of experiments, we assessed whether the 2-D boundary layer could mediate more efficient complex aggregation of antibiotics and thus lead to enhanced signal transduction compared to a 3-D solution. To achieve this, diacetyl vancomycin susceptible receptor (or Ac-VSR)^14^ was added to the solution containing Van or Ori in order to act as a competitor against membrane bound VRR. Van and Ori were chosen because they have consistent binding affinity of ~1 μM^37^ against Ac-VSR and ~4200 μM^37^ against VRR. The concentrations of Van and Ori in solution were kept constant at 50 μM and 0.1 μM respectively. These particular concentrations were chosen because (1) they give rise to relatively large mechanical bending signals, (2) they fall within the linear portions of the Langmuir adsorption isotherms, and (3) they require relatively low levels of Ac-VSR to inhibit the mechanical response at the surface. We therefore reasoned that if the interaction between a drug and Ac-VSR is independent of any surface effects then the amount of Ac-VSR which inhibits the mechanical response at a surface is expected to be the same for both Van and Ori. The concentration of antagonist (Ac-VSR) that gives rise to 50% inhibition (IC_50_) of drug-receptor interactions at the surface was used as measure of membrane effects on the association kinetics and mechanical response. Figure 3c shows the cantilever deflection as a function of Ac-VSR in solution against Van at 50 μM. With increasing levels of Ac-VSR cantilever deflection was found to decrease as a result of the lower levels of free Van molecules available to interact with the membrane bound VRR molecules. The mechanical response with Van was completely inhibited at 500 μM Ac-VSR. With 0.1 μM Ori, however, more than 500 μM of Ac-VSR was required to inhibit the mechanical response which suggests that Ori has a stronger drug-target interaction at the surface than Van (Fig. 3d). The reproducibility of these measurements were tested using four separate chips, and the results are summarized in Fig. 3e,f. To calculate the IC_50_, we analyzed the data using equation (1), whose detailed derivation has been reported previously^14^.


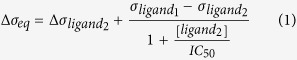


Here σ_eq_ is the equilibrium mechanical stress, σ_*ligand*1_ is the mechanical stress generated by *ligand*_1_ in the absence of a competing *ligand*_2_, σ_*ligand*2_ is the corresponding minimum stress generated by *ligand*_1_ in the presence of a large excess of the concentration of competing *ligand*_2_. The outcome of the fit of equation (1) superimposed onto the mechanical stress data reveal an IC_50_ of 15.4 ± 0.2 μM for Van and 50.7 ± 2.1 μM for Ori (Fig. 3e,f). The IC_50_ for Ori is 3-fold larger than that of Van. The results support the prediction that a 2-D boundary layer is superior to a 3-D solvent in accelerating the association kinetics of ligands thus enabling multivalent interactions that lead to stronger binding and enhanced mechanical force transduction (Fig. 3d).

That a near-membrane layer is critical in driving the recognition and efficacy of drugs at a surface (Fig. 3f), was further confirmed by examining the binding affinity of Van, Rist, CE and Ori to targets in solution and at a surface (Fig. 4a). The binding affinity against VRR in solution was found to be identical for all four antibiotics in the millimolar range. Similarly, with VSR the binding affinity was virtually indistinguishable for all antibiotics in the micromolar range. Remarkably, however, when VRR at a surface was considered, the antibiotics displayed non-uniform binding constants. Moreover, a clear difference was observed between the strength of interactions and ability of antibiotic molecules to polymerise in solution (Fig. 4a). These findings lend support to the hypothesis that a near-membrane layer has a significant influence on molecular recognition and binding efficiency.

### Mechanical force and microbial susceptibility

To investigate whether enhanced antibiotic binding efficiency has any impact on bacterial cell growth, we compared the binding affinity, *K*_*surf*_ or surface thermodynamic equilibrium dissociation constant of drug-target interactions (Supplementary Table S1), to the Minimum Inhibitory Concentration (MIC) values from four different types of bacteria (Supplementary Table S2, S3). Figure 4b shows the logarithmic plots for *K*_*surf*_ versus MICs for all four bacterial samples. The data demonstrated a linear correlation between binding affinity and MICs over a large, 10,000-fold, range. Weak binders were found to have high MICs while those with the strongest binding affinity exhibited lower MIC values. Our results are in agreement with previous findings using SPR measurements whereby vancomycin analogs with differing binding affinities were used to inhibit S. aureus RN4220 (ref. 30).

In order to test the idea that a strong drug binder can generate large-scale mechanical strain on a bacterium surface to impact on its vitality, we examined the mechanical response generated from binding of an antibiotic to its resistant and susceptible targets. We first used slow bacteriostatic Van and rapid bactericidal Ori^38^ against two different bacterial cell wall targets for which Ori and Van concentrations were initially kept constant at 3 μM (Fig. 5a,b). Van was found to show a reduced overall stress transduction against VSR, and no effect on the VRR, compared with Ori, which showed a significantly large mechanical response against both VRR and VSR targets, when measurements were repeated with 3 μM to match the experimental conditions for Van (Fig. 5b). In addition, we found that the buffer washing step alone cannot fully remove successive drug injections, particularly for strong surface binders such as Ori and therefore an additional washing step using 10 mM hydrochloric acid was necessary to regenerate the surface for quantitative analysis. The finding that Ori which tends to polymerise more strongly in solution^37^ generates high mechanical stress against bacterial targets on a cantilever prompted us to repeat the experiments using CE which also has strong complex aggregation ability^37^. The stress transduction of 3 μM CE yielded deflection signals of 20 nm for VRR and 120 nm for VSR targets, respectively (Fig. 6a). These results demonstrate that antibiotics which polymerise strongly in solution are able to interact with both resistant and susceptible targets. To explain the non-bending response observed in Van against the VRR target, we have made the assumption that the complementarities between antibiotics and membrane targets govern molecular recognition to regulate the levels of mechanical signaling. However, the presence of repulsive effects in the oxygen lone pair within the drug’s binding site weakens the Van-VRR bound complex, as well as the deletion of hydrogen bond formation^10^,^15^. Correspondingly, the molecular recognition in the case of CE or Ori against the VRR is also explained as a “lock-key” type binding interaction^39^. However, the enhanced multiple and cooperative receptor-ligand interactions effectively overcome the repulsive effects in the oxygen lone pair^10^,^15^ to increase the mechanical response and bactericidal activity. So far, no experiments have ever successfully demonstrated that Ori can target drug-susceptible and drug-resistant targets with consistently tighter binding interactions. This shows force-based technology plays a key role, and could offer a new way for rapid profiling of clinically important molecules, as well as for sensitive characterization of the relative efficacy of drugs.

To further examine the role of a strong drug binder on the mechanical strain, we repeated the experiments using Van, Rist, CE and Ori at a concentration of 10 μM. This particular concentration was chosen because it gives a saturation stress signal against VSR and is within the clinically relevant range of antibiotic dose (3–27 μM)^40^. Figure 6b demonstrates that a relatively large mechanical response is obtained for each antibiotic against VSR where the mean compressive surface stress was found to be ~33 ± 3 mNm^−1^. In contrast the compressive surface stress with VRR varied for each antibiotic; Van ~0.4 mNm^−1^, Rist ~2 mNm^−1^, CE ~6 mNm^−1^ and Ori ~23 mNm^−1^ (Fig. 6c). The observed difference in mechanical response between VRR and VSR was confirmed statistically. Using one-way ANOVA (Supplementary Table S4, S5) the difference in the level of mechanical response for VRR against different antibiotics was significant (P < 0.000). However there was no significant difference with VSR (P = 0.059). This was further confirmed using an independent sample t-test in which the mechanical response for each antibiotic against VSR and VRR was found to be significant ((P < 0.001), see Supplementary Table S6). We hypothesize that the reduced mechanical response in VRR could be caused by the repulsive effects in oxygen lone pair conferred by the reprogramming of the terminal alanine amino acid residues of bacterium cell^10^ or by the changes in the phenotypic mechanical plasticity^13^.

**Figure 2:**
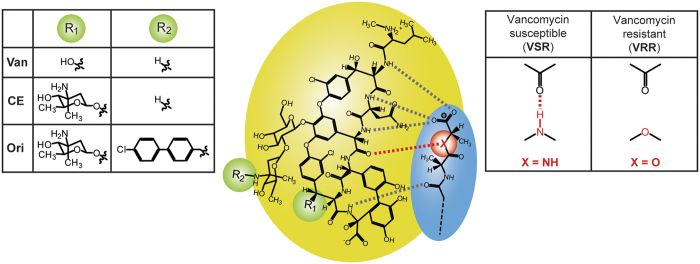
Nanomechanics of drug-target interactions to investigate the impact of remote functional groups on the efficiency of antimicrobial activity. A chemical structure of structurally equivalent peptide backbone of glycopeptide antibiotics (solid yellow circle) showing how they interact with peptide domains of bacterial cell wall amino acid residues (solid blue circle). R groups are used to distinguish the structures of Van, CE, and Ori. The binding of these drugs to a bacterial extracellular target is shown by dotted lines in the antibiotic-peptide complex represented by 5 hydrogen bonds formed upon binding to a drug susceptible target VSR. In VRR phenotype, a single hydrogen bond is deleted from the drug binding pocket distinguishing between vancomycin-susceptible and vancomycin-resistant bacteria. The *X* group (red) on the of drug susceptible phenotype corresponds to a mutation of an amide group to an ester, conferring antimicrobial resistance in vancomycin-susceptible enterococci. The measurements show that chemical *R*_1_ and *R*_2_ groups added at sites remote from the binding pocket could control the mechanical forces induced from drug-target interactions to effect bactericidal activity.

We next determined the critical threshold force sensitivity, *s*, for therapeutically effective drugs such as Ori against drug-susceptible and drug-resistant bacteria. To achieve this, we used the relation *s* = σ_eq_/*n*, where *n* is the number of receptor molecules per cantilever, estimated at ~10^11^ (Supplementary Table S7), and σ_eq_ = 23 mNm^−1^ is the minimum stress responsible for the collective build-up of membrane strain that both drug-resistant and drug-susceptible bacteria cannot ultimately withstand (Fig. 6b,c). By assuming that each drug-target complex acts independently, the deleterious force sensitivity was estimated to be ~20 fNm^−1^. The finding that therapeutically effective drugs such as Ori generate high force sensitivity prompted us to investigate the correlation between mechanical force and microbial susceptibility. We quantified the mechanical force arising from binding of drugs to their bacterial targets on a cantilever (Fig. 6a–c) using the equation *F* = *k*Δz, where Δz is the cantilever deflection signal and *k* is its nominal spring constant of 0.02 Nm^−1^. Figure 6d shows the force generated with VSR and VRR using four different cantilever chips and more than 30 measurements plotted against the MICs obtained from vancomycin-susceptible enterococci and vancomycin-resistant enterococci. An excellent linear correlation was observed between mechanical force and MIC (Fig. 6d). The MIC of Van against MSSA bacteria was 0.67 μM and is consistent with the ~3 nN mechanical force generated against VSR on the cantilever (Fig. 6b). In contrast, the large ~3.6 nN mechanical force generated by Ori against VSR correlates with the low 0.03 μM MIC value of Ori against MSSA. These results are consistent with the time–kill kinetics which is typically between ~6–24 h for Van, a slow bacteriostatic drug, and 15 min-2 h for Ori, which displays rapid bactericidal activity^38^. Van and Ori have different functional chemical groups *R*_1_ and *R*_2_ located remote from their binding pockets but both share a structurally equivalent peptide backbone. This suggests that enhanced bactericidal action can be effected by modifying drug molecules with additional functional groups (Fig. 2). The consistency between the bacterial cell assays and cantilever based measurements strongly suggest that large-scale mechanical forces may play a vital role in bactericidal activity.

### Modelling mechanisms of antibiotic activity

Thus far, we have discussed the impact of near-membrane layer on the association kinetics, mechanical force transduction, specificity of molecular recognition and microbial susceptibility. To gain mechanistic insight (Fig. 7a,b), we averaged measurements for each drug-target interaction over four separate chips and the results are summarized in Fig. 7c,d. To determine the equilibrium dissociation constant *K*_*surf*_, quantitative assays as shown in Fig. 7c,d were determined under steady-state equilibrium conditions for a range of concentrations for each drug-receptor combination. The mechanical signaling response follows an s-shaped curve undergoing its steepest rise at ***N*** ~1/*K*_*surf*_ before reaching a plateau when the binding sites are fully occupied (Fig. 7c,d). To calculate *K*_*surf*_, we used equation (2)^14^, based on the conventional Hill adsorption model^41^.


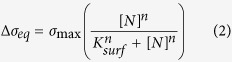


Here, *K*_*surf*_ is raised to the power *n* to ensure that it maintains the dimension of concentration as *n* varies. Setting *n* = 1 yields the Langmuir isotherm where the reactions occur independently, while *n* > 1 corresponds to positive co-operative polyvalent interactions and *n* < 1 is a measure of negative co-operativity. In Fig. 7c,d, we find that the mechanical response at VSR levels off at 50 μM concentration for all antibiotics. In contrast, for the drug-resistant VRR a concentration of more than 1000 μM of Van, Rist or CE was required to reach indistinguishable maximum signals. The outcome of the fit using three parameters (*n, K*_*surf*_ and σ_max_) is summarized in Supplementary Fig. 2. The calculated *K*_*surf*_ for Ori binding to VSR is 40 ± 10 nM, which is 25 times stronger than for Van where *K*_*surf*_ is 1.0 ± 0.3 μM. The *K*_*surf*_ of Ori against VRR is 70 ± 10 nM, an astonishing 11,000 times stronger than for Van where *K*_*surf*_ is 800 ± 300 μM. As a further measurement control and to confirm the accuracy of surface binding affinity, we used a commercially available SPR method (Fig. 7e,f and Supplementary SPR sensor chip functionalization procedure), where the detection of ligands is at a single planar metal surface^30^. The calculated *K*_*surf*_ of 1.0 ± 0.3 μM for Van is indistinguishable from the SPR response-based *K*_*surf*_ value of 1.1 ± 0.4 μM, which suggests that the mechanical assays give the same biophysical parameters as gold-standard biophysical methods.

Next, we analyzed the underlying Hill coefficient *n* of each drug/receptor pair and found that *n* ≤ 1 for Van and Rist, while n > 1 for CE and Ori (Supplementary Table S1), implying that as long as *K*_3_ > *K*_2_ for the latter drugs, the surface interaction is polyvalent. However, the model Hill equation is purely phenomenological and so cannot account for the interrelation of solution and surface influences on the catalytic cycle of multiple interactions. To gain mechanistic insight, we solved the equilibrium equations for Model I (see, Case I, Supplementary Information) to obtain


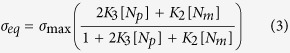


Here *K*_2_ is the surface ligand-target binding strength when ligand molecules follow a monovalent binding mechanism and *K*_3_ is the corresponding constant if they undergo multiple interactions where the dimensionality of the association constants in equation (3) is the inverse of the dissociation constants in equation (2). *N*_*m*_ and *N*_*p*_ are the concentrations of monovalent and polyvalent ligands in solution described by


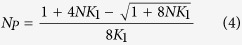



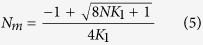


Inspection of equations (3,4,5) shows how multivalent and cooperative effects arise - the Taylor expansion of equations (3) and (4) will add higher order terms, with positive coefficients in *N* to the numerator and denominator of equation (3) provided *K*_3_ > *K*_2_, meaning that fits to equation (2) will yield Hill coefficients *n* > 1. Accordingly, we modeled the entire stress data using equation (3) with 3 fitting parameters (σ_max_, *K*_2_ and *K*_3_) for each membrane receptor, while fixing *K*_1_, to the tabulated values (Supplementary Table S1). For algebraic convenience all the equilibrium constants *K*1–*K*6 in the models and equations below are in the form of association constants (1/M) while the plotted and tabulated values are shown in the form of disassociation constants (M). The maximum mechanical stress σ_*max*_ was fixed at 33.9 ± 3 mNm^−1^ for VSR and 19.2 ± 2 mNm^−1^ for VRR, respectively (Fig. 7c,d, Supplementary Fig. 2 and Supplementary Table S8), because of the simplifying hypothesis, which states that the mechanical saturation stresses for ligand complexes should depend only on the matching of a docking site to an incoming ligand. Moreover, our recent work^34^ has shown the practical need for this hypothesis - when the surface coverage is low, the stress contribution is insufficient to allow independent determination of σ_max_ and *K*_surf_ values. The outcome of the fit of equation (3) is summarized in Fig. 7c which shows good fit to the VSR data. The remarkably good agreement for VSR shows for the first time that an exactly solved equilibrium model with only a single control parameter, *K*_1_, can account for the full variability in the activity of antibiotics against drug-susceptible targets. However, Model I fails dramatically for the VRR data (Fig. 7c) – the “best” fit gives a variability (determined by *K*_1_) in the half-height point of the Langmuir curves of only two orders of magnitude, while the experimental variability is four orders of magnitude.

There are various possible explanations for the failure of Model I. The most trivial, not entailing any surface effects apart from those in the 3-D solution, is that multivalent interactions produce different mechanical stresses than monovalent binding – this translates mathematically into a formula identical to equation (3), but with different weightings of *N*_*m*_ and *N*_*p*_ in the numerator. Our attempt to fit the data with this generalization was unable to account for the much larger variability in *K*_1_ of the VRR data. As an admittedly non-unique attempt to improve on Model I, but in direct response to the strongly implied importance of the surface effects in mediating cooperative processes discussed above, we included a near-membrane layer between the 2-D surface and 3-D solution, yielding Model II (see, Case II, Supplementary Information). This approach allows monovalent and multivalent ligands in solution to enter and exit a near-membrane layer, either to the surface bound states or back to solution, at rates characterized by the interconversion equilibrium constant *K*_4_, within a membrane layer. The additional equilibrium equations were solved analytically to yield


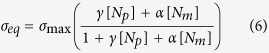


here


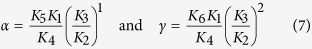


The terms *K*_1_–*K*_6_ are the equilibrium constants defined in Fig. 7b. The identical form of equations (3) and (6) show that *α* and *γ* are renormalized binding coefficients for monovalent and multivalent interactions. It is possible that the aggregation of antibiotic monomers near the interface is facilitated by attractive forces, such as induced dipole interaction and charges. Thus, the difference in the free energy of binding may be caused by the changes in the dimensionality, as well as any other effects that could arise from a constrained environment. The standard approach for translating 3-D binding constants in solution to 2-D membrane environment was first proposed by Bell^42^ and modified by Honig and colleagues^43^. We note that in Bell’s model the possibility that there could be a transition from monovalent to polyvalent interactions at a near-membrane layer even when there is none in solution was not considered, so we have simply generalized Bell’s equation to the form *K*_4_ = *hK*_1_ + *K*_0_, where *K*_0_ is the membrane receptor’s own contribution to multiple interactions and *h* is the inverse of the length representing the effective thickness (transverse to the surface) of near membrane layer to relate the solution and surface reactions. Moreover, given that the molecules studied differ only in the chemical functionality of groups remote from the ligand binding site, and that the tracking of either monovalent or multivalent ligand complexes at the near-membrane layer ought not depend strongly on these groups, the equilibrium constants in equation (7) are combined to produce the constants *C*_*α*_ = (*K*_5_(*K*_3_/*K*_2_)^1) and *C*_*γ*_ = (*K*_6_(*K*_3_/*K*_2_)^2), which again are unlikely to depend strongly on the chemical functionality of remote groups. Consequently, equation (7) is solved to obtain a simplified form for *α* and *γ* in equation (8).


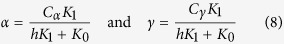


Further work using more microscopic techniques such as X-ray scattering and NMR will be required to understand the individual contributions of these equilibrium constants and to validate the simplifying assumptions made above.

**Figure 3:**
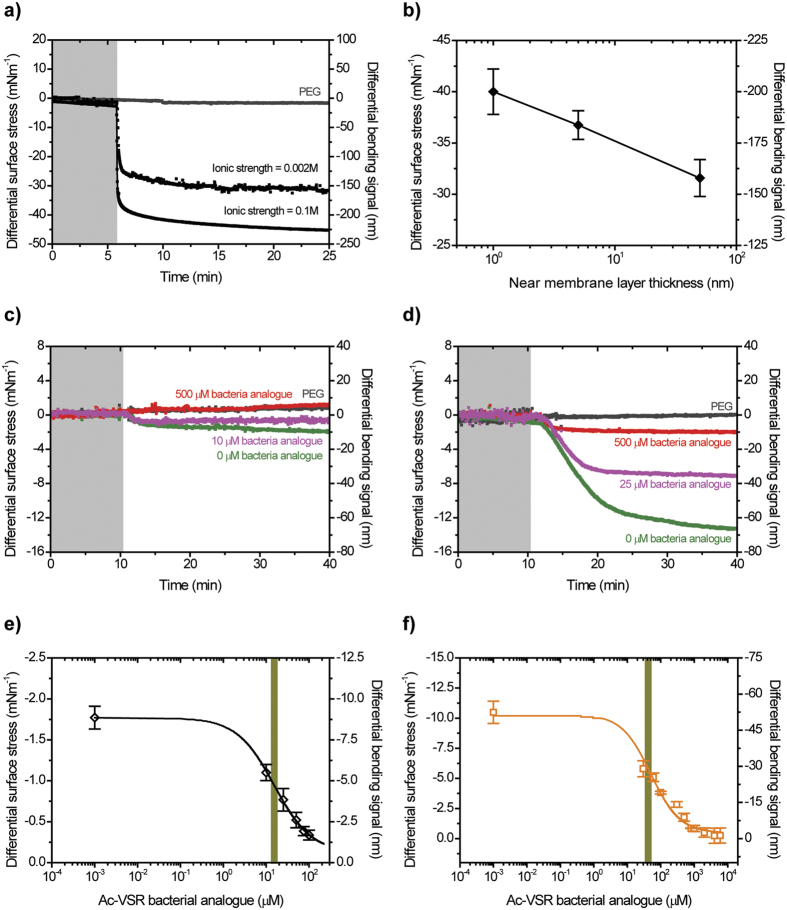
Effect of near-membrane layer on antibiotic binding efficiency. (**a**) Differential cantilever bending signal monitored as a function of ionic strength of 0.002 M (black) and 0.1 M (black) of Van initially fixed at 1000 μM in sodium phosphate buffer solution. (**b**) Semi-logarithmic plot showing measured differential surface stress response for Van (solid diamond square symbols in black) at a total solution concentration fixed at 1000 μM against near membrane layer thickness. The solid line is not a fit but a guide to the eye. (**c**) Differential bending signal of 50 μM Van against VRR receptor in 0 μM (green), 10 μM (magenta) and 500 μM (red) Ac**-**VSR. (**d**) The corresponding differential bending signals of 0.1 μM Ori against VRR receptor in 0 μM (green), 25 μM (magenta) and 500 μM (red) Ac-VSR. In (**a**,**c**,**d**) The grey shaded areas represent the injection of sodium phosphate buffer without drugs lasting for 5 or 10 minutes to establish a baseline and the differential PEG reference signal is shown in grey black. (**e**) Semi-logarithmic plot showing the measured differential stress response for VRR membrane receptors as a function of Ac-VSR concentrations in solution, superimposed on the results of the fit according to Eq. (1) (black solid line) for Van (open black diamond symbols) with the fitting parameters *σ*_ligand1_, *σ*_ligand2_ and IC_50_. (**f**) Semi-logarithmic plot showing the measured differential stress response for VRR membrane receptors as a function of Ac-VSR concentrations in solution, superimposed on the results of the fit according to Eq. (1) (orange solid line) for Ori (open orange square symbols) with the fitting parameters *σ*_ligand1_, *σ*_ligand2_ and IC_50_. In (**e**,**f**) dark yellow shaded area shows where the concentration of IC_50_ is evaluated while in (**b**,**e**,**f**) the surface-stress-data error bars are determined as the standard deviation of surface-stress-data from four separate cantilever chips. These measurements show the role of near membrane layer in effecting the activity of drugs.

**Figure 4:**
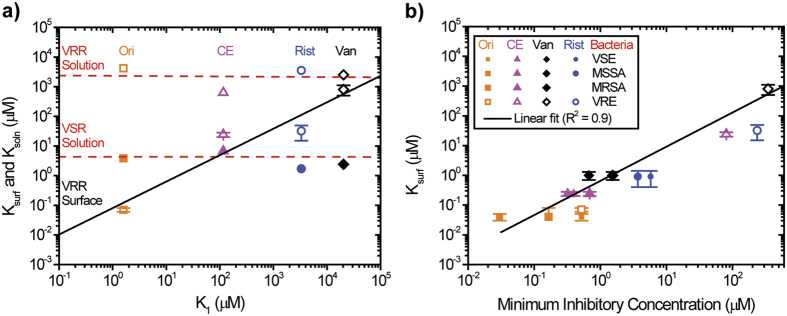
Impact of the strength of drug-target interactions on microbial susceptibility. (**a**) Double logarithmic plots of cantilever surface equilibrium dissociation constants *K*_*surf*_ obtained from antibiotic interactions against VRR at a surface (open symbols) as well as the solution equilibrium dissociation constant, *K*_sol_ for the antibiotic interactions against VRR in solution (open symbols) and VSR (filled solid symbols) as a function of complex aggregation constants, *K*_1_. (**b**) Double logarithmic plot of *K*_*surf*_ versus experimentally determined Minimum Inhibitory Concentration (MIC) values of drug-susceptible intact live bacteria (filled solid symbols with varying sizes) and drug-resistant intact live bacteria (open symbols with varying sizes). In (**a**,**b**) The data are described by equation *K*_surf_ = c(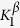) (black solid lines) where ***c*** is a constant of proportionality and β ~ 0.9 ± 0.2 is the power law and *K*_sol_ = *K*_1_ (red dotted lines). The surface stress data error bars were determined as the standard deviation of the surface stress data fitted measurements from four separate cantilever chips. The results show that by considering measurements which quantify near membrane layer and solution effects, we can unlock the connections between specificity of interaction of antibiotics and the corresponding mechanics of bacterial cell walls’ targets responsible for the destruction of bacteria.

**Figure 5:**
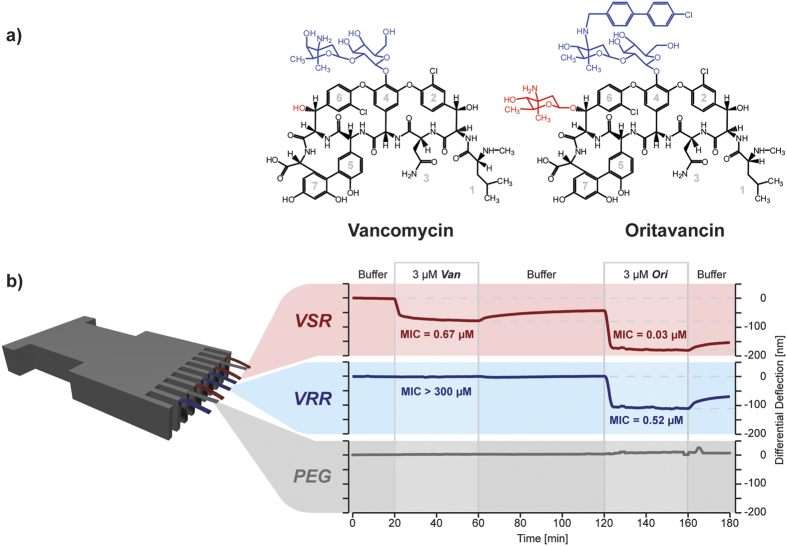
Implications of molecular recognition on mechanical signaling. (**a**) Schematic representation of the chemical structures of Van and Ori drug molecules. For oritavancin, we show that it is the additional sugar group (red) and biphenyl group (blue) residues that contribute to its effectiveness in contrast to Van against vancomycin-resistant bacteria. In (**a**) the numbers 1–7 represent the position of amino acid sequences in the structurally equivalent peptide backbone of vancomycin family of antibiotics. (**b**) Schematic representation of an array of eight rectangular silicon cantilevers, each measuring 500 μm long, 100 μm wide and 1 μm thick. The differential bending signals in buffer of VSR (red) and VRR (blue) are shown upon injection of; buffer washing step, 3 μM Van, buffer washing step and 3 μM Ori and again buffer washing step to remove the drug. Here we show that the buffer washing step alone cannot fully remove successive drug injections particularly for strong surface binders such as Van against VSR and so an additional washing step using 10 mM hydrochloric acid is necessary to regenerate the surface for further use. The MIC values are included for the drug-susceptible (red text) and drug-resistant targets (blue text) to demonstrate the power of cantilever assays in predicting the efficacy of drugs under identical conditions. The findings in effect imply that modestly sized drug molecules such as Ori could be designed to successfully target drug-susceptible and drug-resistant bacteria in equal measure.

**Figure 6:**
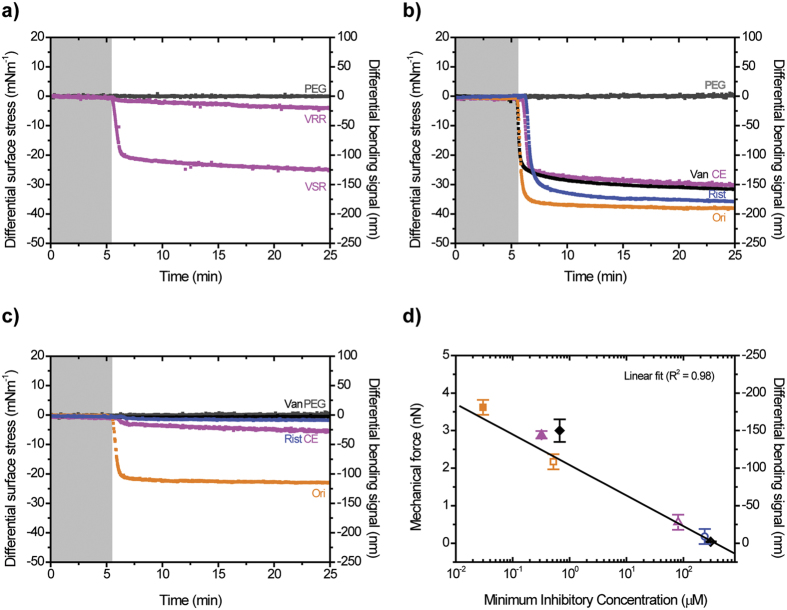
Investigating impact of mechanical forces on microbial susceptibility. (**a**) Differential bending signals in buffer of VSR (magenta) and VRR (magenta) upon injection of 3 μM CE. (**b**) The differential bending signals in buffer for VSR signals upon injection of antibiotics initially fixed at 10 μM of Van (black), Rist (blue), CE (magenta) and Ori (orange). (**c**) The corresponding differential bending signals upon injection of antibiotics initially fixed at 10 μM of Van (black), Rist (blue), CE (magenta) and Ori (orange) against VRR receptors. In (**a**–**c**) downward differential bending signals correspond to compressive (repulsive) surface stress and the differential PEG reference signal is shown in grey black while the shaded areas represent the injection of sodium phosphate buffer without antibiotics lasting for 5 minutes to establish a baseline. (**d**) Measured mechanical forces from drug-target interactions against experimentally determined Minimum Inhibitory Concentration (MIC) values from intact live bacteria of vancomycin-susceptible enterococci (solid symbols) and vancomycin-resistant enterococci (open symbols). The force-data error bars were determined as the standard deviation of force-data from four separate cantilever chips. The measurements suggest that optimization of mechanical forces induced from drug-target interactions could significantly improve the efficacy of antibiotics.

**Figure 7:**
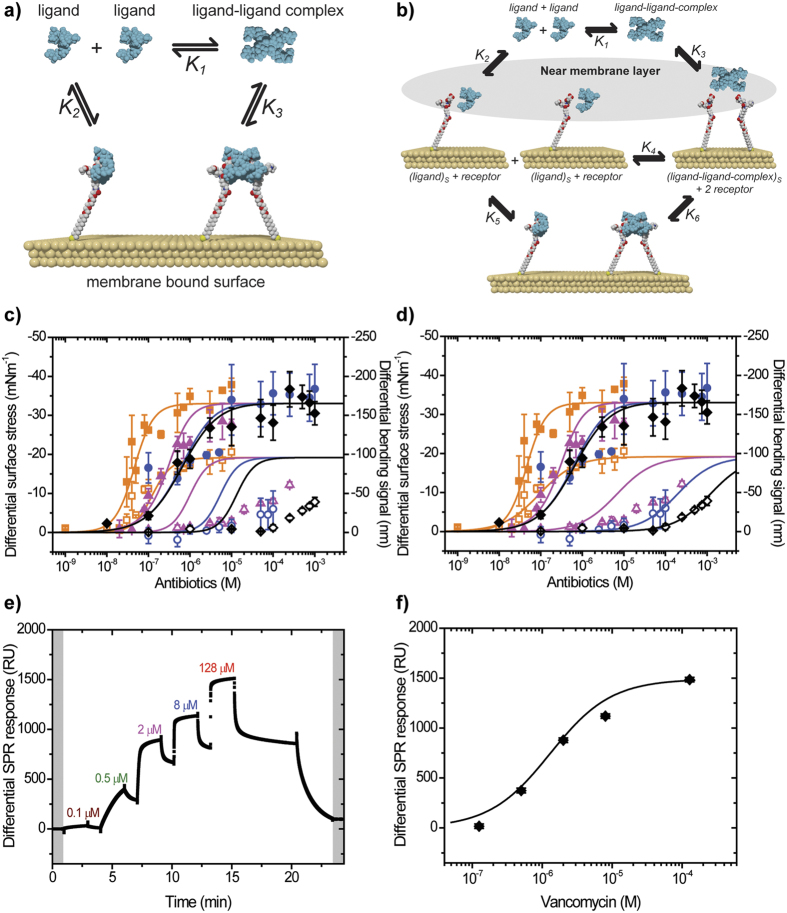
Modelling mechanisms of antibiotic activity. (**a**) Schematic representation of the simple model (I). (**b**) Schematic representation of surface model (II). In (**a**,**b**) K_1_, K_2_, K_3_ K_4_, K_5_ and K_6_ are the thermodynamic equilibrium dissociation constants between aggregation of ligands in solution (K_1_); between solution ligands such as antibiotics and the surface receptor (K_2_); between multivalent ligand complex and the surface receptor (K_3_); between complex aggregation within a near membrane layer (K_4_); between near membrane layer monovalent ligand complexes and the surface receptor (K_5_); and between near membrane layer multivalent ligand complexes and the surface receptor (K_6_). (**c**) Semi-logarithmic plot showing measured differential surface stress response for VSR and VRR targets as a function of antibiotic concentration in solution, [*N*] superimposed on the results of the global fit according to equation (3) (solid lines) derived from model (I). (**d**) Corresponding semi-logarithmic plot showing measured differential surface stress response for VSR and VRR targets as a function of antibiotic concentration in solution, [*N*] superimposed on the results of the global fit according to equation (6) (solid lines) derived from model (II). (**e**) Differential surface plasmon resonance (SPR) response signals for 0.1 μM (wine), 0.5 μM (green), 2 μM (magenta), 8 μM (blue) and 128 μM (red) Van against VSR to demonstrate that cantilever methodology gives the same biophysical surface binding affinity, *K*_*surf*_ as gold-standard methods. (**f**) Semi-logarithmic plot showing the measured differential SPR response signal as a function of Van (black filled diamond symbols) concentrations in solution, superimposed on the results of the fit according to Eq. (2) (solid line) to calculate *K*_*surf*_. In (**b**,**e**) shaded areas represent the near membrane layer and the injection of sodium phosphate buffer without Van lasting for 2 min to establish a baseline with an additional buffer washing step to remove ligands for further use. The study shows that efficacy of drugs against drug-resistant targets can benefit from inherent polyvalent interactions.

In light of Model II, we fitted equations (6) and (8) to antibiotic stress data with 5 parameters (σ_max_, *C*_*α*_, *C*_*γ*_, *K*_0_ and *h*), where *K*_1_ was fixed to the tabulated values (Supplementary Table S1). In Fig. 7d, we show that after accounting for the surface effects, there are improvements to the fits in both the VSR and VRR data, although we observed a less than perfect fit in the case of CE VRR data. The quantitative results from this analysis are consistent with the global fits of the antibiotic stress data using Model II to both VSR and VRR surface targets, revealing *K*_0_ ~ 4 nM for VRR and *K*_0_ ~ 30 M for VSR and *h* = 0.011, common to both surface targets (Supplementary Table S8). The very large *K*_0_ (i.e. *K*_0_ ≫ *hK*_1_), implies that the surface effects for VRR data cannot be neglected. In contrast, for VSR, the very small *K*_0_ (i.e. *K*_0_ ≪ *hK*_1_) suggests that it is the solution rather than the surface effects that dominate the recognition process, in good agreement with equation (3). Although a good fit does not prove that a model is correct, there are several reasons to believe that the proposed model is likely to represent an accurate approximation of the role a membrane bound receptor plays in driving the binding activities. First, the binding parameters obtained in Models I and II are the same in case of VSR data. Second, the idea that the contribution of membrane binding is a cooperative process as determined by the Hill equation is consistent with the proposed model. Third, there is consistency from both quantitative and qualitative perspectives for the membrane reactions as evidenced by the experimental data. Finally, the fact that the competitive inhibition via Ac-VSR is much weaker for Van than Ori (Fig. 3c–f) shows clearly that it is near-membrane rather than solvent effects that are dominant factors in determining the pharmacological efficacy of newer bactericidal drugs such as Ori.

## Discussion

Our experiments have shown unequivocally the importance of surface effects in the mediation of molecular cooperativity and the understanding of antibiotic’s action, furthermore we have made significant steps in unraveling the mechanisms by which a near membrane surface layer regulates molecular association kinetics both for mechanical force transduction and microbial susceptibility. Indeed, even though it is widely accepted that vancomycin analogues inhibit peptidoglycan biosynthesis without substrate binding^44^, here we show that such binding can also contribute to enhanced bactericidal activity of drugs. We find that while a single site binding mechanism in a pre-existing ensemble of conformational states dominates the behavior of susceptible targets, it fails dramatically for drug-resistant targets. However, by including solvent interactions and membrane effects, we demonstrate by comparing an exactly solvable model to our data that the formation of polyvalent interactions catalyzed by the surface itself can account for the enhanced *K*_*surf*_ of Ori on drug-resistant targets of 70 ± 10 nM, an astonishing 11,000 times stronger than for Van where the *K*_*surf*_ is 800 ± 300 μM. This finding is in agreement with solid-state NMR, which has concluded that Ori is a more potent inhibitor of the transpeptidation process than transglycosylation^45^.

The molecular binding events occurring between antibiotics in solution and at membrane bound targets were found to enhance the binding efficiency and mechanical stress signals. Our results from intact bacterial cell assays and stress measurements suggest that bactericidal activity is linked to the extent of molecular recognition and associated mechanical force transduction. We find four orders of magnitude of bactericidal activity are strongly linked to mechanical force, consistent with the picture that following the application of a lethal dose, the insertion of antibiotic molecules into bacterium cell walls can induce a local strain, which grows as the number of reacted regions grows, until a deleterious strain is generated which the bacteria cannot ultimately withstand (Fig. 1c). Consequently, this weakens the overall mechanical strength required to counteract high internal osmotic pressure, leaving bacterium cells susceptible to lysis and death. In summary, we show that by developing the principles^46^,^47^ underlying the interactions of drugs in solution and at a surface, it will be possible to provide more mechanistic insight to fully establish the link between the perturbations observed here and the mode of microbial killing activity. Our findings will aid in the rational design of novel molecules that could be further optimized by using numerical modeling prior to synthesis to ensure that they can successfully target drug-susceptible and drug-resistant bacteria in equal measure. Beyond our key results that near-membrane effects are very important to pharmacological research, our systematic experiments on model cell wall motifs provide a new framework for understanding and eventually designing devices for rapid quantitative screening, as well as for assessing the relative efficacy of drugs. This should be generally applicable to problems in biochemical signaling and pharmacology where the target molecules form relatively dense networks on cell surfaces.

## Methods

### Cantilever measurement procedure

The modified cantilever chip was mounted in a sealed liquid cell. The alignment of each light source onto the free end of each cantilever was confirmed by heating liquid chamber to 1 °C rise. All eight Au-coated cantilever arrays were found to undergo compressive downward bending because of the bimetallic effect caused by the differences in expansion rates of silicon and Au. Care was taken so that the optical alignment error was less than 5% between the minimum and maximum bending signals within the cantilever arrays. In addition, the resonant frequency of all eight Au-coated cantilever arrays was measured to ensure that the variation of spring constant between each cantilever within a chip was ≤1%. The absolute deflection at the free-end of each cantilever ∆*z*_abs_ was measured using a time-multiplexed optical detection system in different liquid environments (Scentris, Veeco Instruments Inc., Santa Barbara, CA, USA). The desired flow rate was first determined before each experiment to ensure a constant flow rate for all the arrays. The data acquisition was automated using LabView (National Instruments Co., Austin, TX, USA) software via a 6-way valve (Serial MVP, Hamilton, Reno, NV, USA). All cantilever bending signals were acquired under an average liquid flow rate of 30–180 μL min^−1^. The equilibrium differential cantilever bending signals were not found to be significantly dependent on the direction or fluid flow rates. The measurement protocol involved the following steps, (1) sodium phosphate buffer (pH 7.4, 0.1 M) for 5 or 10 minutes to establish a baseline; (2) injection of antibiotics in sodium phosphate buffer for 30–60 minutes; (3) to dissociate the complex, sodium phosphate buffer (pH 7.4, 0.1 M) wash for 10–30 minutes; (4) a further washing step using 10 mM HCl for another 30 minutes to regenerate the membrane surface targets; and (5) finally another sodium phosphate buffer step for 10 minutes to restore the baseline signal. We investigated the impact of surface regeneration by undertaking repeated regeneration measurements with more than 10 cycles using 10 mM hydrochloric acid and found that the quality of sensing layers can deteriorate if a large number of receptor regeneration steps are employed particularly when using acidic reagents, thus limiting the lifetime of the sensor itself. The detection specificity was achieved by performing measurements in differential mode, where the bending response of the reference PEG-coated cantilevers, was subtracted from VSR- and VRR-coated cantilever signals.

### Data analysis and statistical characterization

To convert the bending signals into differential surface stress, the raw data from each set of separate cantilever chips were were analyzed off-line using automated data software to rapidly analyze large data sets and remove user bias. The bending signals were converted into differential surface stress between the upper and lower sides of the cantilever, according to the Stoney’s equation


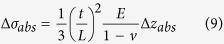


where L is the effective length of the cantilever, *t* is the thickness, *E*/(1 − *ν*) = 181 GPa is the ratio between the Young’s modulus *E* and Poisson ratio *ν* of Si (100) and Δz_abs_ is the absolute cantilever bending deflection. The differential equilibrium surface-stress Δσ_eq_ was calculated by subtracting *in-situ* reference Δσ_abs_ (PEG) surface-stress signal from the absolute stress signals of Δσ_abs_(VSR and VRR coated cantilevers). When comparing four different arrays, there was a high reproducibility of *within-array* measurements, and an increased variance associated with *between-array* measurements in agreement with our previous findings^8^. In this manuscript, a positive absolute deflection signal corresponds to downward bending of the cantilevers due to compressive surface stress and a negative absolute deflection to the upward bending of cantilevers due to a tensile surface stress. The differential stress measurements are typically associated with multiple parameters including number of measurements, concentration and the number of cantilever chips. The statistical analysis was performed by using IBM SPSS Statistics software (IBM Corporation) and the results are summarized in the Supplementary Table S4, S5 and S6.

### Surface plasmon resonance (SPR) measurement procedure

The SPR sensor chip functionalization procedure is summarized (see methods & materials Supplementary Information) while the measurement protocol has been described previously^34^. In brief we used a T100 BIAcore SPR instrument in which a series of binding analyses for Van was performed. Figure 7e,f shows the differential SPR signal in which the responses increases with increasing Van concentration. The corresponding SPR response signals featured an S-shaped curve, with a steep rise, then a plateau when Van concentration was increased beyond 50 μM (Fig. 7e,f). The resulting SPR differential response signals in sodium phosphate buffer solution for 0.1 μM, 0.5 μM, 2 μM, 8 μM and 128 μM were then computed using equation (2) to calculate the surface thermodynamic equilibrium dissociation constant, *K*_*surf*_ of drug-target interactions.

## Additional Information

**How to cite this article:** Ndieyira, J. W. *et al*. Surface mediated cooperative interactions of drugs enhance mechanical forces for antibiotic action. *Sci. Rep.* **7**, 41206; doi: 10.1038/srep41206 (2017).

**Publisher's note:** Springer Nature remains neutral with regard to jurisdictional claims in published maps and institutional affiliations.

## Supplementary Material

Supplementary Information
