# Corrigendum: Surface mediated cooperative interactions of drugs enhance mechanical forces for antibiotic action

**DOI:** 10.1038/srep43980

**Published:** 2017-03-23

**Authors:** Joseph W. Ndieyira, Joe Bailey, Samadhan B. Patil, Manuel Vögtli, Matthew A. Cooper, Chris Abell, Rachel A. McKendry, Gabriel Aeppli

Scientific Reports 7:Article number: 4120610.1038/srep41206; published online: 02 03 2017; updated: 03 23 2017

This Article contains errors. An additional affiliation for Samadhan B. Patil was omitted. The correct affiliations for this Author are listed below:

Departments of Medicine, UCL Institute for Liver and Digestive Health, Royal Free Hospital, London NW3 2QG, UK.

London Centre for Nanotechnology and Departments of Medicine and Physics, University College London, 17-19 Gordon Street, London, WC1H 0AH, United Kingdom.

Manuel Vögtli is incorrectly listed as being affiliated with ‘Departments of Medicine, UCL Institute for Liver and Digestive Health, Royal Free Hospital, London NW3 2QG, UK’. The correct affiliation is listed below:

London Centre for Nanotechnology and Departments of Medicine and Physics, University College London, 17-19 Gordon Street, London, WC1H 0AH, United Kingdom.

Rachel A. McKendry is incorrectly listed as being affiliated with ‘Departments of Medicine, UCL Institute for Liver and Digestive Health, Royal Free Hospital, London NW3 2QG, UK’. The correct affiliation is listed below:

London Centre for Nanotechnology and Departments of Medicine and Physics, University College London, 17-19 Gordon Street, London, WC1H 0AH, United Kingdom.

