# Supplementary material for: Surface mediated cooperative interactions of drugs enhance mechanical forces for antibiotic action
Source: Sci Rep. 2017 Feb 3;7:41206. doi: 10.1038/srep41206 (PMC5290737; doi:10.1038/srep41206)
Supplement: Supplementary Information [file srep41206-s1.pdf]

# Surface mediated cooperative interactions of drugs enhance mechanical forces for antibiotic action

Joseph W. Ndieyira, Joe Bailey, Samadhan B. Patil, Manuel Vögtli, Matthew A. Cooper, Chris Abell, Rachel McKendry and Gabriel Aeppli

**Supplementary Table S1: The calculated thermodynamic and nanomechanical parameters for each antibiotic-receptor complex using equation (2), where *Ksurf* is the surface equilibrium dissociation constant and *n* is the Hill coefficient compared with the reported values from solution assays and SPR methods.**

| Antibiotics | Type | Hill coeff. (*n*) | $K_{surf}$ Cantilever (µM) | $K_{surf}$ SPR (µM) | $K_{surf}$ Solution (µM) | $K_1$ Solution (µM) |
|---|---|---|---|---|---|---|
| Van | VSR | 1.0 ± 0.2 | 1.0 ± 0.3 | 1.1 ± 0.4 | 1.0 (ref. 1) | 20000 ref. 2 |
| | VRR | 1.2 ± 0.2 | 800 ± 300 | 526 (ref. 3) | 2400 (ref. 1) | |
| Rist | VSR | 0.6 ± 0.3 | 0.9 ± 0.5 | 0.2 (ref. 4) | 1.7 (ref. 1) | 3000 ref. 5 |
| | VRR | 0.7 ± 0.2 | 32 ± 17 | 6.7 (ref. 5) | 3850 (ref. 6) | |
| CE | VSR | 1.3 ± 0.3 | 0.24 ± 0.04 | 0.3 (ref. 3) | 1.0 (ref. 7) | 120 ref. 2 |
| | VRR | 1.5 ± 0.5 | 24 ± 4 | 3.2 (ref. 4) | 4100 (ref. 8) | |
| Ori | VSR | 1.8 ± 0.4 | 0.04 ± 0.01 | n/a | 3.8 (ref. 2) | 1.6 ref. 2 |
| | VRR | 2.7 ± 0.6 | 0.07 ± 0.01 | n/a | 4200 (ref. 2) | |

n/a $K_{surf}$ value not available.

**Supplementary Table S2: The Minimum Inhibitory Concentration (MIC) values for each antibiotic against clinical isolates of methicillin-susceptible *Staphylococcus aureus* (MSSA) ATCC 29213. MIC values are given in standard mg/l units and then converted into molar concentrations**.

| Antibiotics | MIC (mg/l) | MIC (µM) |
|---|---|---|
| Van | 1.0 | 0.67 |
| Rist | 8.0 | 3.69 |
| CE | 0.5 | 0.32 |
| Ori | 0.06 | 0.03 |

**Supplementary Table S3: The corresponding MICs in Mueller-Hinton and Broth Turbidity Inspection values for each antibiotic against clinical isolates of vancomycin resistant E. *faecalis* (VRE), methicillin-resistant Staphylococcus *aureus* (MRSA), vancomycin susceptible E. *faecalis* (VSE) strains (assays conducted in the absence of polysorbate-80). MIC values are given in standard mg/l units**.

| **Antibiotics** | ***VRE* (mg/l)** | ***VSE* (mg/l)** | **MRSA (mg/l)** |
|---|---|---|---|
| Van | 512 (ref. 2) | 0.5-4 (ref. 9) | 0.5-4 (ref. 9) |
| Rist | 512 (ref. 5) | 12.5 (ref. 10) | n/a |
| CE | 128 (ref. 5) | 0.25-1 (ref. 9) | 0.16-0.63 (ref. 9) |
| Ori | 0.25-2 (ref. 9) | 0.25-2 (ref. 9) | 0.13-1.01 (ref. 9) |

n/a MIC value not available.

**Supplementary Table S4: One-way ANOVA statistical analysis of maximum stress data for drug-resistant (VRR) target exposed to antibiotics**.

| Antibiotic | Chips | Mean | Std. Deviation | Std. Error | 95% Confidence Interval for Mean Lower Bound | 95% Confidence Interval for Mean Upper Bound | F | Sig. |
|---|---|---|---|---|---|---|---|---|
| Van | 4 | 7.4 | 1.7 | 0.8 | 4.7 | 10.1 | | |
| Rist | 4 | 7.5 | 2.1 | 1.1 | 4.1 | 10.9 | | |
| CE | 4 | 11.5 | 2.8 | 1.4 | 7.1 | 15.9 | | |
| Ori | 4 | 20.6 | 3.0 | 1.5 | 15.8 | 25.4 | | |
| Total | 16 | 11.8 | 5.9 | 1.5 | 8.6 | 14.9 | 25.6 | .000 |

**Supplementary Table S5: One-way anova statistical analysis of maximum stress data for drug-susceptible (VSR) target exposed to antibiotics**.

| Antibiotic | Chips | Mean | Std. Deviation | Std. Error | 95% Confidence Interval for Mean Lower Bound | 95% Confidence Interval for Mean Upper Bound | F | Sig. |
|---|---|---|---|---|---|---|---|---|
| Van | 4 | 33.5 | 5.5 | 2.73 | 24.8 | 42.2 | | |
| Rist | 4 | 34.5 | 4.5 | 2.3 | 27.3 | 41.7 | | |
| CE | 4 | 27.0 | 1.8 | 0.9 | 24.2 | 29.8 | | |
| Ori | 4 | 33.8 | 2.5 | 1.3 | 29.8 | 37.8 | | |
| Total | 16 | 32.2 | 4.7 | 1.2 | 29.7 | 34.7 | 3.3 | .059 |

**Supplementary Table S6: Independent samples t-test for equality of means for drug-susceptible (VSR) and drug-resistant (VRR) targets exposed to antibiotics**.

| VSR | VRR | Chips | Mean difference | Std. Error | Sig. | 95% Confidence Interval Lower Bound | 95% Confidence Interval Upper Bound |
|---|---|---|---|---|---|---|---|
| Van | Van | 4 | 26.1 | 2.9 | .000 | 19.1 | 33.1 |
| Rist | Rist | 4 | 27.0 | 2.5 | .000 | 20.9 | 33.2 |
| CE | CE | 4 | 15.5 | 1.6 | .000 | 11.5 | 19.5 |
| Ori | Ori | 4 | 13.2 | 1.9 | .001 | 8.4 | 18.1 |

**Supplementary Table S7: Summary of normalized sulphur to gold peak intensities acquired at a take-off angle of 90$^o$ for each self-assembled monolayer (SAM) compared with reference PEG to calculate the footprint of VSR and VRR receptor molecules respectively**.

| Description | Surface area per molecule (Å$^2$) |
|---|---|
| PEG | 27 (ref. 11) |
| VSR | 44 ± 6 |
| VRR | 41 ± 6 |

**Supplementary Table S8: The calculated nanomechanical parameters for VSR and VRR data using model (II)**.

| Receptor | Description | $\sigma_{max}$ (mNm$^{-1}$) | $C_\alpha$ (nM) | $C_\gamma$ (nM) | $h$ | $K_0$ (M) |
|---|---|---|---|---|---|---|
| VSR | all | 33 ± 3.0 | 5.6 x $10^4$ | 58.8 | 0.011 | 33 |
| VRR | all | 19.2 ± 2.0 | 0.3 | 0.1 | 0.011 | 4.3 x $10^{-9}$ |

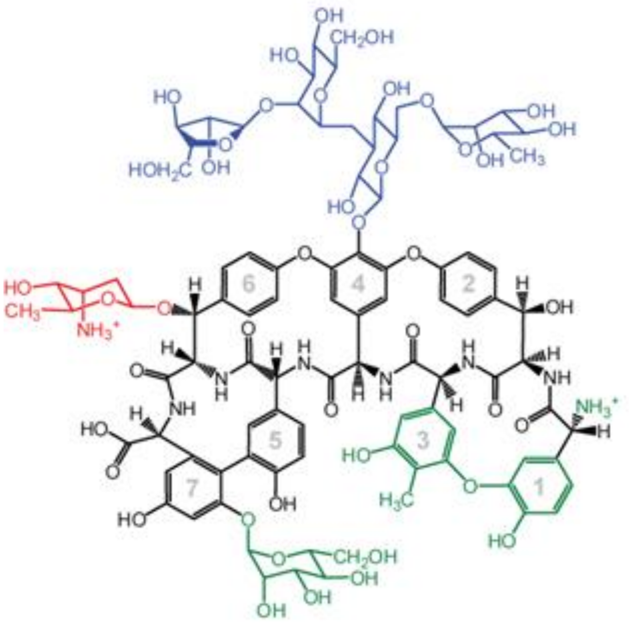

**Supplementary Fig. 1**: **The chemical structure of ristomycin in which the difference to vancomycin side groups is highlighted in red and green. The residue numbers of the seven amino acid backbone are displayed in grey**.

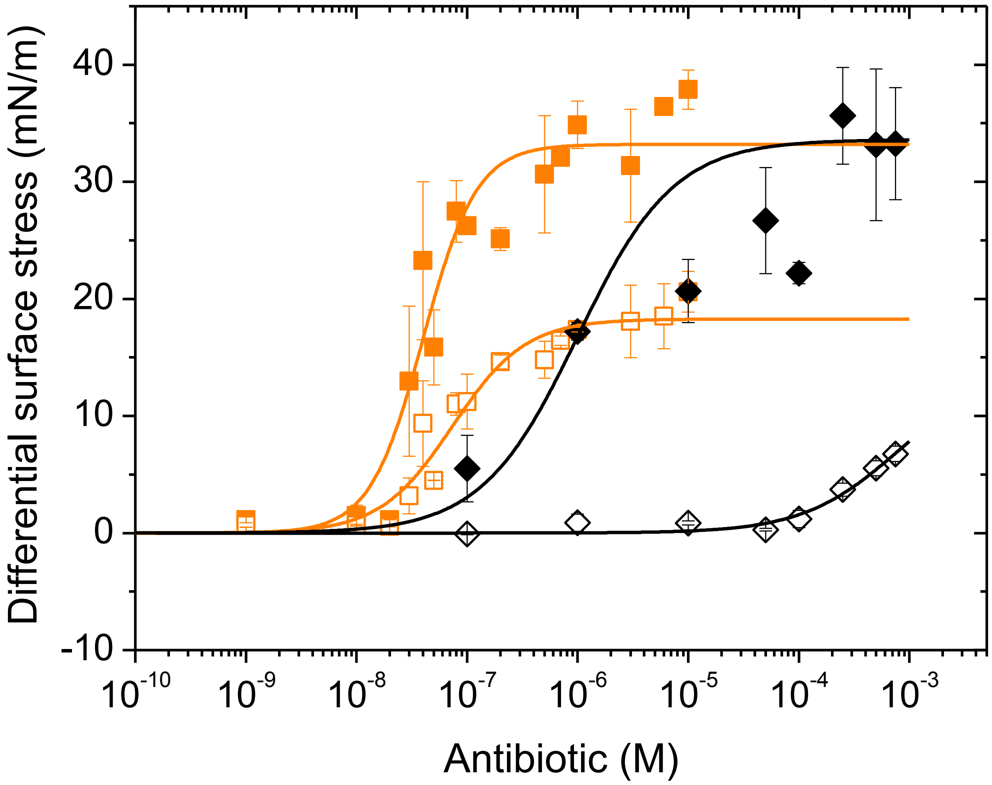


**Supplementary Fig. 2**: **Investigating nanomechanics of drug-target interactions using multiple cantilever arrays**. Semi-logarithmic plot showing measured differential surface stress response for VSR (orange and black solid symbols) and VRR (orange and black open symbols) against Van and Ori concentrations in solution, superimposed on the results to equation (2) (solid lines) to calculate *Ksurf*. We show the injection of vancomycin and oritavancin only, though similar measurements were performed for ristomycin and chloroeremomycin.

## METHODS & MATERIALS

**Outline of the chemical synthesis of thiolated mucopepides.** The synthesis of extracellular targets found in bacteria cell envelopes, herein termed vancomycin-susceptible receptor (or VSR) and the reprogrammed version of VSR termed vancomycin-resistant receptor (or VRR) is described in ref. 12. The cleaved products were purified by reverse phase HPLC by varying the mobile phase from 5% to 95% of acetonitrile in water (with 0.5% trifluoroacetic acid) and characterized using NMR and HRMS.

**Determination of MIC values to methicillin-susceptible *Staphylococcus aureus* (MSSA).** Minimum Inhibitory Concentration (MIC) is defined as the lowest concentration of antibacterial agent required to inhibit growth of a microorganism after incubation. The antibiotics vancomycin (Van) and ristomycin (Rist) were obtained from Sigma-Aldrich while chloroeremomycin (CE) and oritavancin (Ori) were obtained from the Medicine Company Inc., U.S.A. To prepare antibiotic stock solutions for MICs assay, 5 mg/ml of each drug was dissolved in water containing an appropriate volume of 0.002% polysorbate-80 or PS80 obtained from Sigma-Aldrich which corresponds to 20 μM to eliminate nonspecific interactions of drugs to surfaces particularly for Ori which is known to bind to glass and plastics[13]. Microbiological broth with PS80 maintained at 20 μM was used in the drug dilution steps until the required final (assay) concentration. Diluted concentrations of each drug were assayed by adding an inoculum of methicillin-susceptible *Staphylococcus aureus* (MSSA) ATCC 29213 cells and the MIC determined by broth microdilution according to Clinical Laboratory Standards Institute (CLSI) methodology M07-A8 (CLSI 2009a)[14]. The results as shown in Supplementary Table S2 for each antibiotic against clinical isolate of MSSA ATCC 29213 strains conducted in the presence of

PS80 are within the quality control ranges defined in the guideline[15]. Table S3 shows the corresponding MICs in Mueller-Hinton and Broth Turbidity Inspection values for each antibiotic against clinical isolates of vancomycin resistant E. faecalis (VRE), methicillin-resistant *Staphylococcusaureus* (MRSA), vancomycin susceptible E. faecalis (VSE) strains from literature (assays conducted in the absence of PS80).

**Chemicals and reagents for stress assays**. Sodium phosphate salts obtained from Sigma-Aldrich were dissolved in 1 litre of ultrapure water using 18.2 MΩ·cm resistivity obtained from Millipore Co., Billerica, MA, U.S.A. The salts were mixed to yield pH 7.4 and filtered using 0.2 µm filters obtained from Millipore. Van, Rist, CE and Ori were dissolved in sodium phosphate buffer containing 0.002% or 20 µM PS80 and maintained at this concentration to minimize Ori loss to surfaces of vessels particularly glass or plastics[13] during the stress assays. For experiments to examine the impact of the ionic strength, freshly prepared buffer solutions at pH 7.4 at four different ionic strengths of $\mu$ = 0.1 M, 0.2 M, 0.02 M and 0.002 M were used to prepare 1000 µM Van stock solutions. The mixtures were gently vortexed for 15 minutes with an additional 5 minutes of sonication to ensure complete solubility. Brief centrifugation of 10 min at 3000 rpm and at temperature of 22$^{o}$ C was necessary to remove air bubbles.

**Cantilever metallisation procedure**. Cantilever arrays, each 1 µm thick, 500 µm long, and 100 µm wide with a pitch of 250 µm, spring constant of 0.02 $Nm^{-1}$ were fabricated by IBM Research Laboratory, Switzerland. These cantilever arrays were cleaned with freshly prepared piranha solution at ratio of 1:1 $H_2SO_4$ and $H_2O_2$ for 20 min, followed by thorough rinsing with ultrapure water. They were transferred into a freshly prepared piranha solution for a further 20 min and

thoroughly rinsed with ultrapure water. After a thorough cleaning with ultrapure water, they were rinsed with pure ethanol and dried on a hotplate at 75 °C to remove any traces of trapped water on each array. The freshly cleaned cantilever arrays were coated on one side with a thin film of 2 nm titanium which acted as an adhesion layer for an additional 20 nm layer of gold (BOC Edwards Auto 500, U.K., vacuum pressure of $10^{-7}$ mbar and evaporation rate, 0.7 nm/s). The titanium and gold film thickness were confirmed by using a quartz crystal monitor placed directly above the target sources.

**Cantilever functionalization procedure**. Glass capillary tubes (King Precision Glass, Claremont, CA, USA) were arranged on a functionalization stage according to the cantilever pitch size of 250 μm, each coated with surface capture molecules selected randomly to avoid user bias. The surface capture molecules contained alkanethiol and formed self-assembled monolayers (SAM). Three types were considered, herein termed VSR and a reprogrammed version of VSR termed VRR as well as a reference alkanethiol terminating in triethylene glycol (PEG) known to resist nonspecific interactions of drug molecules[12]. They were diluted in ethanol solution to yield a total concentration of 1 μM. Care was taken to ensure that each solution of the surface capture molecules was confined onto individual cantilever sensors to avoid or minimize cross-contaminations. The cantilevers were incubated inside the glass capillaries for 20 min, washed three times with ethanol and ultrapure water before use. The uniformity of surface coverage was confirmed by X-ray photoelectron spectroscopy (XPS)[16], (see, Supplementary Table S7).

**Surface plasmon resonance (SPR) sensor chip functionalization procedure**. We applied the same cantilever functionalization procedure to the SPR sensor chip functionalization. The plain Au-coated SPR chips were incubated in 100 μl ethanolic solutions of alkanethiol SAMs of VSR, VRR and PEG at a total concentration of 1 μM for 20 min, washed three times with ethanol and buffer solution before use.

**Statistical data analysis**

The differential stress measurements obtained from cantilever chips are typically associated with multiple parameters including number of repeated measurements, concentration and the number of cantilevers where each array has eight individual cantilevers. The statistical analysis was found to be essential for replacing a vast quantity of data with numbers such as the averages and standard deviations. To obtain the statistical summary of the differential stresses of drug susceptible (VSR) and drug resistant (VRR) targets exposed to antibiotics in each concentration, we employed a range of formulae. To express the arithmetic mean of the differential equilibrium stress data ($\sigma_{\mathbf{eq}}$), all the differential stress data ($\sigma_{\mathbf{diff}}$) in each concentration were added up and then divided by the total number (n) of experiments using the expression

$$\sigma_{eq} = \frac{\sum \sigma_{diff}}{n} \tag{1}$$

To calculate the standard deviation of the stress data ($\sigma$), we used the following expression

$$\sigma = \sqrt{\frac{\sigma_{eq} - \sigma_{diff}}{n-1}} \tag{2}$$

Subsequently, the standard error (SE) was calculated based on the standard deviation of the stress data using the expression

$$SE = \frac{\sigma}{\sqrt{n}} \tag{3}$$

To determine the confidence intervals where the ranges of values include the true distribution of the stress data, we performed statistical analysis using commercial IBM SPSS Statistics software (IBM Corporation) and the results are summarized in the Supplementary Table S4, S5 and S6.

## SURFACE MODELLING

### Case I: Direct surface interactions

We first proposed that solvent effects rather than near-membrane are dominant factors important in determining pharmacological activities of drugs. Accordingly, we considered that molecules can interact with the surface targets without undergoing surface catalyzed polymerization so that the reactions are quantified by considering the distributions between monovalent $[N_m]$ and polyvalent $[N_p]$ ligands in solution. In particular, the concentrations of the ligands in solution are constrained by the condition

$$[N_m] + 2[N_p] = [N] \tag{4}$$

So that

$$[N_m] = \frac{-1 + \sqrt{8K_1[N] + 1}}{4K_1} \tag{5}$$

$$[N_p] = \frac{1 + 4K_1[N] - \sqrt{8K_1[N]+1}}{8K_1} \qquad (6)$$

Here $[N]$ is the total number of molecules in the solution and $K_1$ is the complex aggregation constant where the dimensionality is the inverse of the dissociation constant of the tabulated values in Table S1.

**Single binding mechanism**

For this case, we consider that the empty sites ($n_s - n_o$) on the surface undergo reversible monovalent interactions with ligands in solution. The equation describing the number of ligands engaged in monovalent binding is

$$K_2(n_s - n_o)[N_m] = n_m \qquad (7)$$

Here $n_s$ is the total number of binding sites on the surface, $n_o$ is the total number of surface binding sites occupied by molecules, $n_m$ is the total number of bound monovalent ligands at a surface and $K_2$ is the surface ligand-target binding strength for ligand molecules binding monovalently.

**Multiple binding mechanisms**

For this case, we consider that the empty sites ($n_s - n_o$) on the surface undergo reversible polyvalent interactions with ligands in solution. The equation describing the number of ligands engaged in multivalent binding is

$$K_3(n_s - n_o)[N_p] = n_p \qquad (8)$$

Here $n_p$ is the total number of bound multivalent ligands at a surface and $K_3$ is the surface ligand-target binding strength for ligand molecules binding multivalently.

To quantify surface binding interactions, the concentrations of the ligands at the surface are constrained by the condition

$$n_m + 2n_p = n_o \tag{9}$$

Equations (7), (8) and (9) are solved analytically to yield

$$\sigma_{eq} = \sigma_{\max}\left(\frac{2K_3\left[N_p\right]+K_2\left[N_m\right]}{1+2K_3\left[N_p\right]+K_2\left[N_{\mathrm{m}}\right]}\right) \tag{10}$$

where $\theta = (n_o/n_s)$ is the fraction of the surface occupied by the binding ligands and $\sigma = \sigma_{max}\theta$.

**Case II: Effect of surface on polyvalent interactions**

In this case we propose that the near-membrane layer rather than solvent effects are dominant factors in determining pharmacological activities of drugs. This is because membrane receptors are polyvalent and such polyvalence certainly should contribute to the increased binding affinity when going from solution to surface targets. Thus, the subsequent strengthening of surface interactions can be defined by an additional five equilibrium equations as summarized.

$$K_2(n_s - n_o)_{BL}[N_m] = n_{m\ BL} \tag{11}$$

$$K_3(n_s - n_o)_{BL}[N_p] = n_{p\ BL} \tag{12}$$

$$K_4\left(n_{m\ BL}\right)^2 = n_{p\ BL} \tag{13}$$

$$K_5\left(n_s - n_o\right)n_{m\ BL} = n_m \tag{14}$$

$$K_6\left(n_s - n_o\right)n_{p\ BL} = n_p \tag{15}$$

Here $n_{m\ BL}$ is the total number of monovalent ligands at a surface boundary layer and $n_{p\ BL}$ is the corresponding number if they are polyvalent. $K_4$ is the surface complex aggregation constant. $K_5$ is the surface ligand-target binding strength, when ligand molecules follow monovalent binding mechanism and $K_6$ is the corresponding constant if they undergo multivalent interactions. Equations (11)-(15) can be solved analytically to yield

$$\sigma_{eq} = \sigma_{\max}\left(\frac{\gamma[N_p] + \alpha[N_m]}{1 + \gamma[N_p] + \alpha[N_m]}\right) \tag{16}$$

Here $\alpha$ and $\gamma$ are renormalized binding coefficients when ligand molecules follow monovalent binding or multivalent interactions and are defined as

$$\gamma = \frac{2K_6K_1}{K_4}\left(\frac{K_3}{K_2}\right)^2 \quad and \quad \alpha = \frac{K_5K_1}{K_4}\left(\frac{K_3}{K_2}\right)^1 \tag{17}$$

**Deductions**

If we consider only monovalent binding, equation (17) reduces to

$$\Delta\sigma_{eq} = \sigma_{\max}\left(\frac{\alpha\left[N_{\mathrm{m}}\right]}{1 + \alpha\left[N_{\mathrm{m}}\right]}\right) \tag{18}$$

Equation (18) is a general form of Langmuir Adsorption Isotherm which describes the equilibrium binding interactions of independent species at surface, again validating the accuracy of equations (3) and (6) in the main text.

**Definition of a near membrane layer**

To define a near membrane layer effect, we used the Debye screening theory such that when a charged molecule is placed in a buffer solution, the electrostatic interactions can cause counterions to surround the molecule, partially offsetting the molecular charge. The simple approximation of the resulting screened electric potential from a point charge Q is

$$\Phi = \frac{Q}{4\pi\varepsilon_o d} e^{\frac{-d}{\lambda_d}} \tag{19}$$

where

$$\lambda_d = \frac{1}{\sqrt{4\pi l_B \sum_i \rho_i z_i^2}} \tag{20}$$

In equations (19-20), Φ is the Debye-Hückel model, $Q$ is the screened electric potential from a point charge, $\varepsilon_o$ is the dielectric constant, $d$ is the separation distance between charged solution ligand and the surface receptors, $l_B$ is the Bjerrum length, $\rho_i$ is the ion density, $z_i$ is the valence for ion species $i$ and $\lambda_d$ is the Debye length. Based on the ionic strength of 0.1M of sodium phosphate buffer solution as employed in our experiments and by assuming a fixed charge of surface receptor molecules, a near membrane layer thickness was estimated using equation (20).

## Supplementary references


(1) Nieto, M. & Perkins, H. R. Modifications of acyl-d-alanyl-d-alanine terminus affecting complex-formation with vancomycin. *Biochem. J.* **123**, 773-787 (1971).

(2) Allen, N. E. & Nicas, T. I. Mechanism of action of oritavancin and related glycopeptide antibiotics. *FEMS Microbiol. Rev.* **93**: 511-532 (2003).

(3) Cooper, M. A. Fiorini, M. T. Abell, C. & Williams, D. H. Binding of vancomycin group antibiotics to D-alanine and D-lactate presenting self-assembled monolayers. Bioorg. Med. Chem. **8**, 2609–2616 (2000).

(4) Cooper, M. A., Williams, D. H. & Cho, Y. R. Surface plasmon resonance analysis of glycopeptide antibiotic activity at a model membrane surface. *Chem. Commun.* **17**, 1625-1626 (1997).

(5) Cooper, M. A. & Williams, D. H. Binding of glycopeptide antibiotics to a model of vancomycin-resistant bacterium. *Chem & Biol*. **6**, 891-899 (1999).

(6) O'Brien, S. W. *et al*. Kinetic barriers and ordering of non-covalently bound states. *Org. Biomol. Chem.* **1**, 472-477 (2003).

(7) Mackay, J. P. Gerhard, U., Beauregard, D. A. Maplestone, R. A. & Williams, D. H. *J.* Dissection of the contributions toward dimerization of lycopeptide Antibiotics. *Am. Chem. SOC.* **116**, 4573–4580 (1994).

(8) Dancer, R. J., Try, A. C., Sharman, G. J. & Williams, D. H. *J.* Binding of a vancomycin group antibiotic to a cell wall analogue from vancomycin-resistant bacteria. *Chem. Commun.* **6**, 1445–1446 (1996).

(9) Nicas, T. I. *et al*. Semisynthetic glycopeptide antibiotics derived from LY264826 active against vancomycin-resistant enterococci. *Antimicrob. Agents Chemother*. **40**, 2194-2199 (1996).

(10) McComas, C. C., Crowley, B. M., Hwang, I. & Boger, D. L. Synthesis and evaluation of methyl ether derivatives of the vancomycin, teicoplanin, and ristocetinaglycon methyl esters. *Bioorg. Med. Chem. Lett*. **13**, 2933–2936 (2003).

(11) Schwendel D., Hayashi T., Dahint R., Pertsin A., Grunze M., Steitz R. & Schreiber F. Interaction of water with self-assembled monolayers: neutron reflectivity measurements of the water density in the interface region. *Langmuir*, **19**, 2284-2293 (2003).

(12) Ndieyira, J. W. *et al*. Nanomechanical detection of antibiotic-mucopeptide binding in a model for superbug drug resistance. *Nature Nanotech.* **3**, 691-696 (2008).

(13) Arhin, F. F. *et al*. Effect of polysorbate 80 on oritavancin binding to plastic surfaces: implications for susceptility testing. *Antimicrob. Agents Chemother*. **52**, 1597-1603 (2008).

(14) CLSI 2009a. Methods for Dilution Antimicrobial Susceptibility Tests for Bacteria That Grow Aerobically; Approved Standard Eighth Edition. CLSI document M07-A8. Wayne, PA: Clinical and Laboratory Standards Institute.

(15) CLSI 2009b. Performance Standards for Antimicrobial Susceptibility Testing; Nineteenth Informational Supplement. CLSI document M100-S19. Wayne, PA: Clinical and Laboratory Standards Institute.

(16) Patil, S. B. *et al*. Decoupling competing surface binding kinetics and reconfiguration of receptor footprint for ultrasensitive stress assays. *Nature Nanotech.* **10**, 899–907 (2015).
